# Role of the 5-HT_4_ receptor in chronic fluoxetine treatment-induced neurogenic activity and granule cell dematuration in the dentate gyrus

**DOI:** 10.1186/s13041-015-0120-3

**Published:** 2015-05-15

**Authors:** Yuki Imoto, Toshihiko Kira, Mamiko Sukeno, Naoya Nishitani, Kazuki Nagayasu, Takayuki Nakagawa, Shuji Kaneko, Katsunori Kobayashi, Eri Segi-Nishida

**Affiliations:** Department of Physiological Chemistry, Graduate School of Pharmaceutical Sciences Kyoto University, Yoshida Shimoadachi-cho, Sakyo-ku, Kyoto 606-8501 Japan; Department of Molecular Pharmacology, Graduate School of Pharmaceutical Sciences Kyoto University, Yoshida Shimoadachi-cho, Sakyo-ku, Kyoto 606-8501 Japan; Department of Pharmacology, Graduate School of Medicine, Nippon Medical School, Sendagi, Bunkyo-ku, Tokyo Japan; Japan Science and Technology Agency, Core Research for Evolutional Science and Technology, Saitama, 332-0012 Japan; Department of Biological Science and Technology, Faculty of Industrial Science and Technology, Tokyo University of Science, 6-3-1 Niijuku, Katsushika-ku, Tokyo 125-8585 Japan

**Keywords:** Antidepressant, Neurogenesis, 5-HT_4_ receptor, Hippocampus, Maturation, Granule cell

## Abstract

**Background:**

Chronic treatment with selective serotonin (5-HT) reuptake inhibitors (SSRIs) facilitates adult neurogenesis and reverses the state of maturation in mature granule cells (GCs) in the dentate gyrus (DG) of the hippocampus. Recent studies have suggested that the 5-HT_4_ receptor is involved in both effects. However, it is largely unknown how the 5-HT_4_ receptor mediates neurogenic effects in the DG and, how the neurogenic and dematuration effects of SSRIs interact with each other.

**Results:**

We addressed these issues using 5-HT_4_ receptor knockout (5-HT4R KO) mice. Expression of the 5-HT_4_ receptor was detected in mature GCs but not in neuronal progenitors of the DG. We found that chronic treatment with the SSRI fluoxetine significantly increased cell proliferation and the number of doublecortin-positive cells in the DG of wild-type mice, but not in 5-HT4R KO mice. We then examined the correlation between the increased neurogenesis and the dematuration of GCs. As reported previously, reduced expression of calbindin in the DG, as an index of dematuration, by chronic fluoxetine treatment was observed in wild-type mice but not in 5-HT4R KO mice. The proliferative effect of fluoxetine was inversely correlated with the expression level of calbindin in the DG. The expression of neurogenic factors in the DG, such as brain derived neurotrophic factor (*Bdnf*), was also associated with the progression of dematuration. These results indicate that the neurogenic effects of fluoxetine in the DG are closely associated with the progression of dematuration of GCs. In contrast, the DG in which neurogenesis was impaired by irradiation still showed significant reduction of calbindin expression by chronic fluoxetine treatment, suggesting that dematuration of GCs by fluoxetine does not require adult neurogenesis in the DG.

**Conclusions:**

We demonstrated that the 5-HT_4_ receptor plays an important role in fluoxetine-induced adult neurogenesis in the DG in addition to GC dematuration, and that these phenomena are closely associated. Our results suggest that 5-HT_4_ receptor-mediated phenotypic changes, including dematuration in mature GCs, underlie the neurogenic effect of SSRIs in the DG, providing new insight into the cellular mechanisms of the neurogenic actions of SSRIs in the hippocampus.

## Background

Chronic antidepressant administration increases adult neurogenesis in the dentate gyrus (DG) of the hippocampus [1,2], which is thought to contribute, in part, to antidepressant activity in rodent behavioral models [3]. Antidepressants also regulate the expression of various genes, such as those encoding neurotrophic factors, in the DG [4,5], which is important for behavioral and neurogenic actions of antidepressants in the DG [6]. Among antidepressants, selective serotonin (5-HT) reuptake inhibitors (SSRIs) including fluoxetine specifically increase synaptic 5-HT levels. 5-HT receptors are subdivided into 14 subtypes in rodents [7]; results of recent pharmacological studies have suggested that in addition to the 5-HT_1A_ receptor [3], the 5-HT_4_ receptor [8,9] mediates SSRI-induced neurogenic actions in the DG. It has been reported that 5-HT_4_ receptor agonists such as RS67333 show antidepressant-like behavioral effects in the forced swim test and sucrose consumption test, and enhance neurogenic activity in the DG in rats [8]. A recent study also showed that the 5-HT_4_ receptor antagonist GR125487 partially prevents the neurogenic effects of fluoxetine in a mouse model of anxiety and depression [9]. However, it is still largely unknown how the 5-HT_4_ receptor mediates the neurogenic effect of SSRIs in the DG.

We have recently shown that chronic treatment with fluoxetine transforms mature granule cells (GCs) to an immature-like state in the DG in a so-called “dematuration” process, and that the 5-HT_4_ receptor is essential for this change [10]. Dematured GCs exhibit reduced expression of mature GC markers including calbindin, increased somatic excitability, and reduced activity-dependent synaptic facilitation. Chronic fluoxetine treatment also induces marked enhancement of 5-HT- and dopamine-induced synaptic potentiation at the mossy fiber synapse, the sole output of the GCs [10,11]. These findings give rise to questions regarding the mechanism through which the 5-HT_4_ receptor contributes to both neurogenic and dematuration effects of chronic SSRI administration within the DG and regarding the association between these cellular and phenotypic changes in the DG.

In this study, we address these issues in 5-HT_4_ receptor knockout (5-HT4 KO) mice with the C57BL/6 J background. We demonstrate that the 5-HT_4_ receptor plays an important role in both fluoxetine-induced adult neurogenesis and GC dematuration in the DG, and that these phenomena are closely associated with each other.

## Results

### Requirement of the 5-HT_4_ receptor for fluoxetine-induced neurogenic effects in the DG

Pharmacological studies using 5-HT_4_ receptor agonists and antagonists have suggested that the 5-HT_4_ receptor is involved in the adult neurogenesis of the DG [8,9]. However, there is no study examining the influence of genetic deficiency of the 5-HT_4_ receptor on SSRI-induced neurogenesis in the DG. In this study, we examined the effect of SSRI treatments on adult neurogenesis in 5-HT4R KO mice with the C57BL/6 J background, in which we have previously demonstrated the involvement of the 5-HT_4_ receptor in the fluoxetine-induced dematuration of GCs.

**Figure 1:**
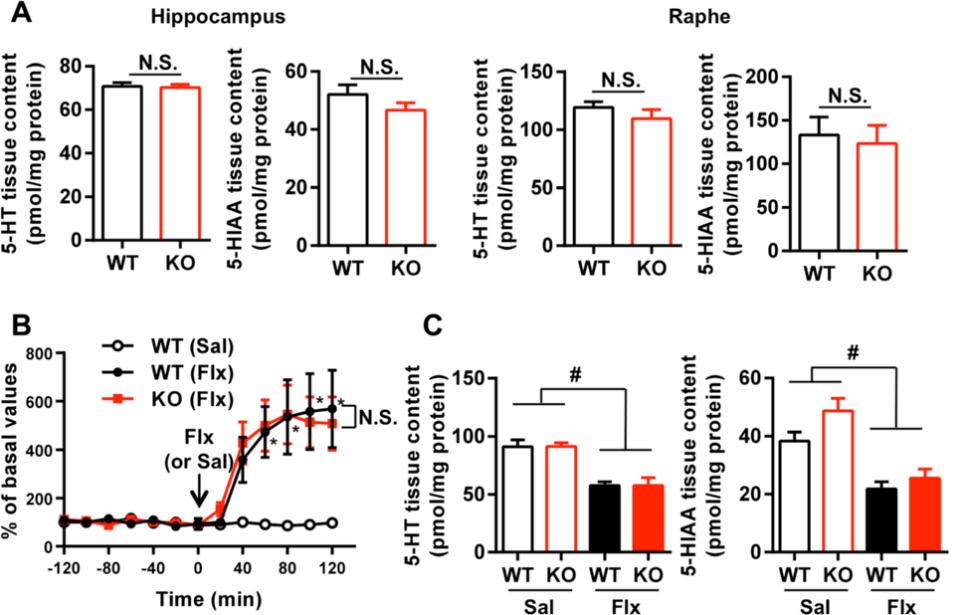
5-HT levels in the hippocampus of 5-HT4R KO mice with the C57BL/6 J-background. **(A)** Tissue 5-HT and 5-HIAA levels in the hippocampus and raphe nuclei. Data are expressed as the mean ± SEM (n = 7 or 8). N.S., not significant for unpaired *t*-tests. **(B)** Fluoxetine (Flx)-induced increase in extracellular 5-HT concentrations in the hippocampus. After equilibration, dialysate samples were collected every 20 min with either fluoxetine (22 mg/kg) or saline (Sal) administered at time zero. The average value of seven basal samples for each animal was defined as 100% and used for normalization. Data are expressed as the mean ± SEM (n = 4 for fluoxetine, n = 3 for saline). Main effect of group: P = 0.0499; main effect of time point: P < 0.0001; interaction of group and time point: P < 0.0001. P value was determined by two-way ANOVA for repeated measures. * P < 0.01 [WT(Sal) vs WT(Flx)] for *post hoc* Bonferroni’s test after two-way ANOVA; N.S, not significant [WT(Flx) vs KO(Flx)] for *post hoc* Bonferroni’s test at every time point after two-way ANOVA. **(C)** Tissue 5-HT and 5-HIAA levels in the hippocampus after chronic fluoxetine treatment. Fluoxetine (22 mg/kg) was administered for 3 weeks. Data are expressed as the mean ± SEM (n = 7 or 8). Main effect of drug: ^#^P < 0.0001 (5-HT), ^#^P < 0.0001 (5-HIAA); main effect of genotype: P = 0.9734 (5-HT), P = 0.0423 (5-HIAA); interaction of drug and genotype: P = 0.9790 (5-HT), P = 0.3243 (5-HIAA); P values determined by two-way ANOVA.

Because it has been reported that the 5-HT content in the raphe nuclei is decreased in a distinct line of 5-HT4R KO mice with the 129/Sv background [12], we first measured the contents of 5-HT and its main metabolite, 5-hydroxyindole acetic acid (5-HIAA), in the hippocampus and the raphe nuclei in 5-HT4R KO mice with the C57BL/6 J background. The contents of 5-HT and 5-HIAA in both the hippocampus and the raphe nuclei were similar between wild-type (WT) and 5-HT4R KO mice (Figure 1A). We also examined the increase in extracellular 5-HT accumulation after a single administration of fluoxetine by *in vivo* microdialysis assays (Figure 1B). Intraperitoneal injection of fluoxetine caused a significantly larger increase in hippocampal 5-HT levels than vehicle injection. The increase in 5-HT levels after administration of fluoxetine was not significantly different between genotypes.

We further examined the contents of 5-HT and 5-HIAA in the hippocampus after chronic fluoxetine treatment. Consistent with a previous report [13], chronic fluoxetine treatment significantly reduced the total contents of 5-HT and 5-HIAA in the hippocampus, which is considered to reflect feedback inhibition of 5-HT biosynthesis in the raphe nucleus. We found that the 5-HT content after chronic fluoxetine treatment were not significantly different between WT and 5-HT4R KO mice (Figure 1C). Therefore, we used this mutant line to address the involvement of the 5-HT_4_ receptor in SSRI-induced neurogenic activity in the DG. However, because the main effect of genotype for 5-HIAA was significant, we could not exclude the possibility that differences in 5-HT metabolism might lead to a higher amount of 5-HIAA in 5-HT4R KO mice to WT mice.

**Figure 2:**
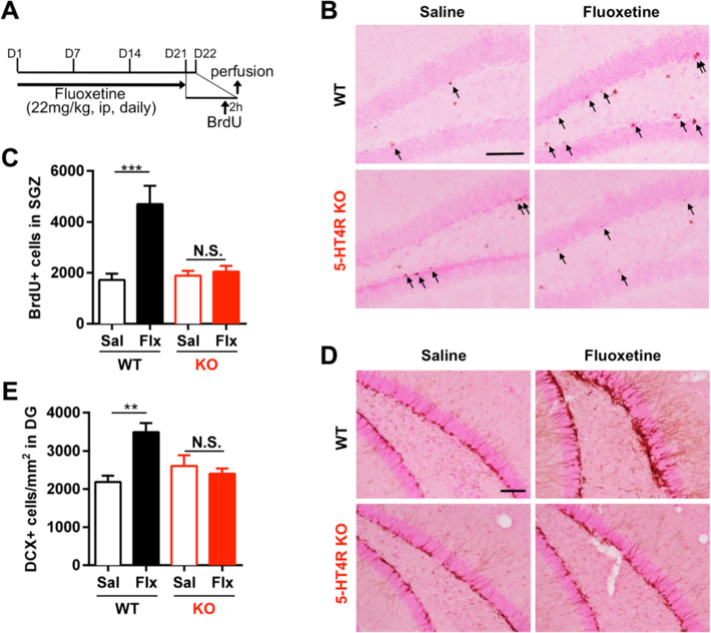
Effect of chronic fluoxetine treatment on adult neurogenesis in 5-HT4R KO mice. **(A)** Experimental scheme. Mice were intraperitoneally (ip) injected with fluoxetine (Flx) at a dose of 22 mg/kg for 21 days and were administered BrdU 24 h after the last treatment (on day-22) at a dose of 150 mg/kg. Mice were sacrificed 2 h after the BrdU injection. **(B)** Immunohistochemical visualization of BrdU in the SGZ of the DG. Scale bar: 100 μm. Arrows represent BrdU-positive cells. **(C)** Quantification of BrdU-positive cells in the SGZ of the DG in WT mice and 5-HT4R KO mice. Data are expressed as the mean ± SEM (n = 4 or 5). Main effect of drug: P = 0.0023; main effect of genotype: P = 0.0107; interaction of drug and genotype: P = 0.0048; P values determined by two-way ANOVA. *** P < 0.001 and N.S, not significant for *post hoc* Bonferroni’s test, respectively, after two-way ANOVA. **(D)** Representative images of anti-doublecortin (DCX) immunohistochemistry in the DG. Scale bar: 100 μm. **(E)** Quantification of DCX-positive immature neurons in the DG of the WT mice and 5-HT4R KO mice. The number of DCX-positive cells in the DG is shown as the number of cells per square millimeter of DG area. Data are expressed as the mean ± SEM (n = 4 or 5). Main effect of drug: P = 0.0214; main effect of genotype: P = 0.1372; interaction of drug and genotype: P = 0.0032; P values determined by two-way ANOVA. ** P < 0.01 and N.S., not significant for *post hoc* Bonferroni’s test, respectively, after two-way ANOVA.

Mice were intraperitoneally treated with 22 mg/kg fluoxetine for 21 days (from day-1 to day-21), and 5-bromodeoxyuridine (BrdU) was given 2 h before sacrifice on day-22 to label proliferating cells (Figure 2A). Chronic fluoxetine treatment significantly increased the number of BrdU-positive cells in the subgranular zone (SGZ) of the DG compared with saline treatment in WT mice, whereas no significant difference was observed between saline and fluoxetine treatments in 5-HT4R KO mice (Figure 2B and C). We next assessed the number of immature neurons by immunostaining for doublecortin (DCX), a marker of neurogenesis (Figure. 2D and E). The number of DCX-positive cells in the DG was significantly increased in fluoxetine-treated WT mice, whereas no significant difference was observed between saline and fluoxetine treatments in 5-HT4R KO mice. These results demonstrate that the 5-HT_4_ receptor is important for the neurogenic effect of chronic fluoxetine treatment in the DG.

### The 5-HT_4_ receptor is expressed in the mature GCs of the DG

**Figure 3:**
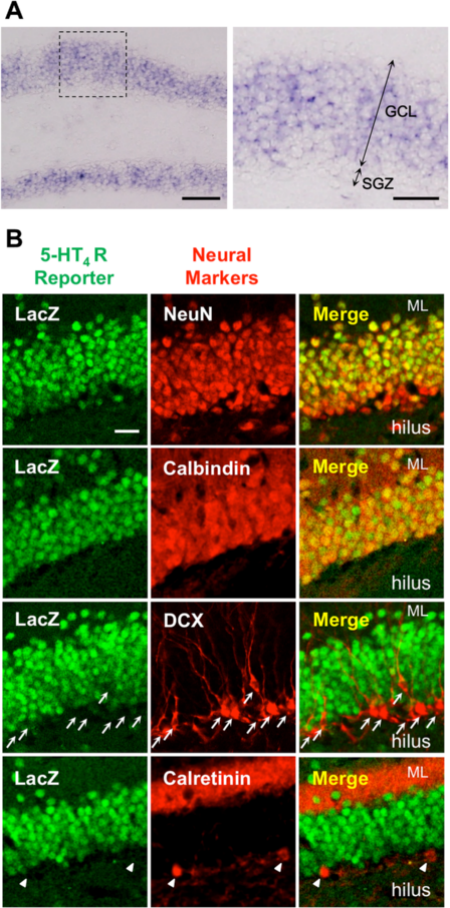
Expression of the 5-HT_4_ receptor in the DG. **(A)** *in situ* hybridization analysis of 5-HT_4_ receptor mRNA expression. GCL: granular cell layer, SGZ: subgranular zone. Scale bars: 100 μm (left) and 30 μm (right). **(B)** Representative images of β-galactosidase (LacZ) and markers of granule cells; NeuN, calbindin, doublecortin (DCX), and calretinin. Scale bar: 20 μm. Arrows represent DCX-positive cells. Arrow heads represent calretinin-positive cells.

It has been reported that the 5-HT_4_ receptor is abundantly expressed in the DG [14,15]. As reported previously, we observed that 5-HT_4_ receptor mRNA (*Htr4*) is mainly expressed in the granule cell layer, rather than in the SGZ, of the DG by *in situ* hybridization (Figure 3A). The expression level of *Htr4* in the DG was not significantly changed by chronic fluoxetine treatment (control, 1.000 ± 0.091; fluoxetine, 1.348 ± 0.152; [fold change], P = 0.079, unpaired Student’s *t*-test; assessed by quantitative reverse transcription (RT)-PCR; n = 6 each). To determine the cell types expressing the 5-HT_4_ receptor in the DG, we examined β-galactosidase immunoreactivity (LacZ-IR) in the hippocampus of the 5-HT4R KO mice, in which the β-galactosidase gene was ‘knocked-in’ at the *Htr4* gene locus. We found that the LacZ-IR was co-localized with the immunoreactivity of a neural marker, NeuN, and that of a marker for mature GCs, calbindin. However, they were not co-localized with the immunoreactivity of DCX, a marker of neural progenitors and immature neurons, and that of calretinin, a marker of immature GCs, in the SGZ (Figure 3B), suggesting that the 5-HT_4_ receptor is mainly expressed in mature GCs.

### The neurogenic effect of fluoxetine is correlated with dematuration of GCs in the DG

**Figure 4:**
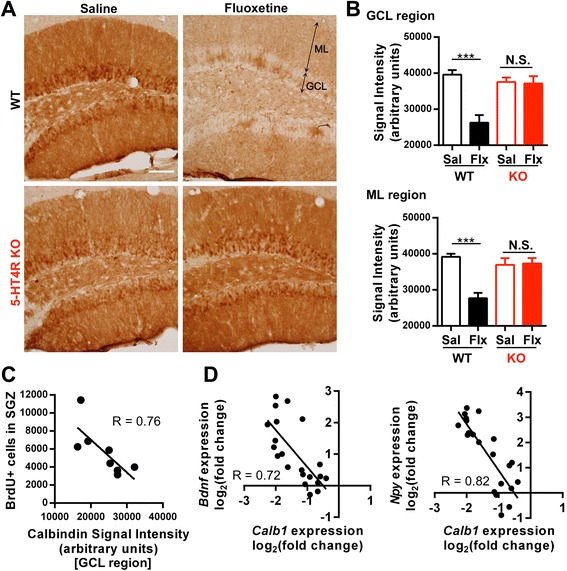
Correlation between enhancement of adult neurogenesis and dematuration of GCs. **(A)** Representative images of calbindin-IR. GCL: granule cell layer, ML: molecular layer. Scale bar: 100 μm. **(B)** Quantification of calbindin-IR in the GCL and the ML in WT mice and 5-HT4R KO mice. Data are expressed as the mean ± SEM (n = 4 or 5). Main effect of drug: P = 0.0014 (GCL), P = 0.0015 (ML); main effect of genotype: P = 0.0237 (GCL), P = 0.0208 (ML); interaction of drug and genotype: P = 0.0022 (GCL), P = 0.0009 (ML); P values determined by two-way ANOVA. *** P < 0.001 and N.S., not significant for *post hoc* Bonferroni’s test, respectively, after two-way ANOVA. **(C)** Comparison between the number of BrdU-positive cells in the SGZ and calbindin-IR intensity of the granule cell layer in WT mice. Mice that received fluoxetine treatment (22 mg/kg) for 3 or 4 weeks were plotted in the analysis. The Pearson correlation coefficient (R) was calculated (P = 0.0278). **(D)** Comparison of gene expression changes after chronic fluoxetine treatment between calbindin (*Calb1*) and *Bdnf* or *Npy*. Gene expression was normalized by the average gene expression in control mice. Mice received fluoxetine treatment (22 mg/kg) for 3 or 4 weeks. Four independent experiments were included in the analysis. The Pearson correlation coefficient (R) was calculated (*Bdnf* vs. *Calb1*, P = 0.0002, *Npy* vs. *Calb1*, P < 0.0001).

We previously reported that the 5-HT_4_ receptor is necessary for fluoxetine-induced dematuration in GCs [10]. Dematured GCs exhibit various phenotypic changes including reduced expression of mature GC markers, increased somatic excitability, and reduced activity-dependent synaptic facilitation. Because the expression of the 5-HT_4_ receptor in the DG is mainly localized to mature GCs, we hypothesized that the progression of GC dematuration via the 5-HT_4_ receptor is correlated with the neurogenic effect of fluoxetine within the SGZ of the DG. To evaluate the phenotypic changes related to GC dematuration, the reduction in the expression level of the mature GC marker calbindin was used as an index of dematuration of GCs [10]. Chronic fluoxetine treatment significantly reduced calbindin-IR in both the granule cell layer and molecular layer of the DG in WT mice, but not in 5-HT4R KO mice (Figure 4A and B). To examine the correlation between the proliferative and dematuration-inducing effects of fluoxetine, we compared the number of BrdU-positive cells and the calbindin-IR in the DG of the same animal, and found that the number of proliferating cells was closely and inversely associated with the calbindin-IR in the granule cell layer (GCL) (Figure 4C). It is known that antidepressant treatment increases the expression of growth factors and neuropeptides including *Bdnf* and neuropeptide Y (*Npy*) in the DG [4,5], which are suggested to be involved in the enhanced neurogenesis [16-18]. We also examined the correlation between the increased expression of these factors and reduced calbindin mRNA (*Calb1*) in the individual fluoxetine-treated DG (as compared to average control DG mRNA levels); the expression changes, as determined by quantitative RT-PCR, were inversely correlated (Figure 4D). The combined observation of inverse correlations between the intensity of calbindin-IR and cell proliferation, along with the expression of neurogenic factors, respectively, suggest that the neurogenic effect of fluoxetine in the SGZ is closely associated with the progression of GC dematuration in the DG.

### Dematuration of GCs by chronic fluoxetine treatment still occurs without adult neurogenesis

**Figure 5:**
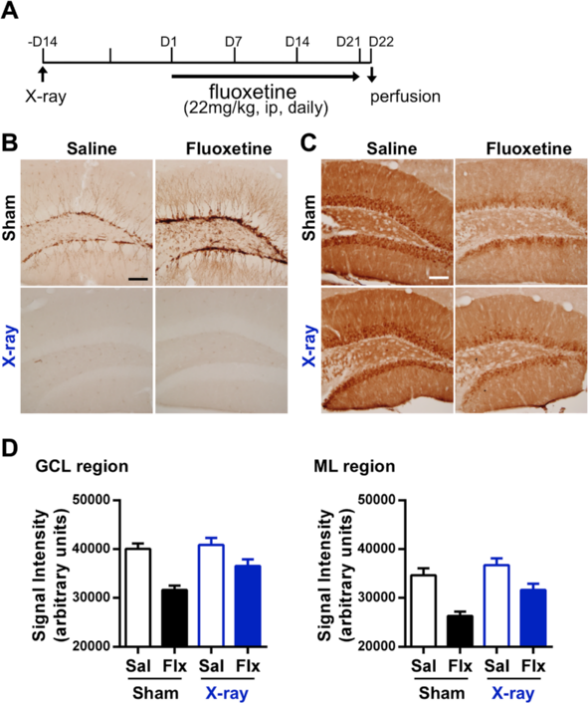
Effect of X-ray irradiation on fluoxetine-induced adult neurogenesis and dematuration in the DG. **(A)** Experimental scheme. Mice were irradiated with X-rays (10 Gy) 14 days before initiation of fluoxetine treatment (ip, intraperitoneally injected). Mice were sacrificed 24 h after the final drug treatment. **(B** and **C)** Representative images of DCX (B) and calbindin **(C)** immunohistochemistry. Scale bars: 100 μm. **(D)** Quantification of calbindin-IR signal intensity in the GCL and the ML in control (Sham) and X-ray-irradiated mice. Data are expressed as the mean ± SEM (n =5 or 6). Main effect of drug: P < 0.0001 (GCL), P < 0.0001 (ML); main effect of X-ray irradiation: P = 0.0303 (GCL), P = 0.0087 (ML); interaction of drug and X-ray irradiation: P = 0.1089 (GCL), P = 0.2085 (ML); P values determined by two-way ANOVA.

We next examined whether adult neurogenesis affects the dematuration of GCs by fluoxetine. To address this point, we used X-ray irradiation to block the production of new neurons within the SGZ, and examined the effect of fluoxetine on the reduction of calbindin-IR in the neurogenesis-depleted DG. Mice were irradiated with X-rays directed at the whole cranium 14 days before initiation of the fluoxetine treatment (Figure 5A). We confirmed that X-ray irradiation dramatically depleted the DCX-positive cells in the DG of both saline- and fluoxetine-treated mice (Figure 5B). Irradiation by X-rays had no effect on calbindin-IR in saline-treated mice (Figure 5C and D). Compared to saline, chronic fluoxetine treatment also reduced calbindin-IR in X-ray-irradiated mice (Figure 5C and D). These results suggest that neurogenesis is not a critical factor for the dematuration of GCs by fluoxetine treatment.

## Discussion

**Figure 6:**
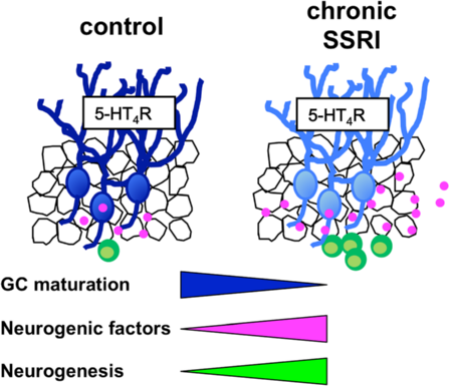
Model of 5-HT_4_ receptor-mediated increased neurogenesis and GC dematuration in the DG induced by chronic SSRIs. Chronic SSRI treatment induces dematuration of GCs and increases adult neurogensis via the 5-HT_4_ receptor (5-HT_4_R) in the DG. Dematured GCs exhibit an increase in expression of neurogenic factors, which may be involved in enhanced adult neurogenesis in the SGZ of the DG.

We have previously shown that chronic SSRI treatment reverses the mature phenotype of GCs to an immature like-state, called dematuration, via the 5-HT_4_ receptor [10]. In this study, we demonstrated that the 5-HT_4_ receptor plays an important role in the fluoxetine-induced increases in both cell proliferation and DCX-positive cells in the DG. This increase in DCX-positive cells may be due to either the development of additional neuronal progenitors or the dematuration of mature GCs. We also demonstrated that fluoxetine-induced increases in cell proliferation and the expression of neurogenic factors in the DG are closely associated with the progression of GC dematuration (Figure 6).

### Involvement of 5-HT receptors in the neurogenic effect of fluoxetine

Previous reports showed that 5-HT receptor subtypes other than the 5-HT_4_ receptor contribute to the effect of chronic fluoxetine treatment in the DG. One candidate is the 5-HT_1A_ receptor. 5-HT_1A_ receptor KO mice did not exhibit cell-proliferating effects of SSRI treatment [3]. Although it is known that desensitization of the 5-HT_1A_ autoreceptor in serotonergic neurons after chronic SSRI treatment is a key factor in enhanced serotonergic synaptic transmission, the role of the 5-HT_1A_ receptor in the hippocampus has also been suggested to be necessary for antidepressant responses. The 5-HT_1A_ receptor is abundantly expressed in the DG [14], and its activation increases cell proliferation in the DG even in the 5-HT-depleted rats [19]. These studies suggest that the 5-HT_1A_ receptor is involved in neurogenesis mediated by SSRIs. However, it was recently reported that 8-OH-DPAT—a 5-HT_1A_ receptor agonist—did not increase cell proliferation in the DG in the C57BL/6 mice [20]. This suggests that the involvement of the 5-HT_1A_ receptor in neurogenesis may be different among species and stains and that the contribution of the 5-HT_4_ receptor to SSRI-enhanced neurogenesis may be more prominent in the C57BL/6 strain.

Similarly, there are two possible pathways mediating the 5-HT4 receptor action in the neurogenic effects of fluoxetine: regulating the activity of serotonergic neurons and activating the neurogenic signals in hippocampus. It has been reported that the activity of serotonergic neurons in the dorsal raphe nuclei is increased by 5-HT_4_ receptor activation [21]. In fact, the 5-HT level is reduced in the dorsal raphe nuclei in 5-HT4R KO mice of the 129/Sv background [12]. However, the present study showed that 5-HT and 5-HIAA tissue contents, in addition to extracellular 5-HT accumulation induced by fluoxetine in the hippocampus, were not different between WT and 5-HT4R KO mice of the C57BL/6 J background (Figure 1). This result indicates that the deficits of cellular responses in the DG to fluoxetine in 5-HT4R KO mice described in this study were not due to a change in 5-HT level. However, we cannot exclude the possibility that the activity of serotonergic neurons in the dorsal raphe nuclei is regulated by the 5-HT_4_ receptor in a manner that does not depend on 5-HT levels in the hippocampus.

### Interaction between increased neurogenesis and dematuration of GCs in the DG

In this study, we showed that the neurogenic effect of fluoxetine in the DG is closely associated with the progression of GC dematuration in the same animals (Figure 4). This association is supported by the fact that fluoxetine-induced neurogenesis and GC dematuration showed a similar dose and time dependency in C57BL/6 background mice [10,22,23], and both effects of fluoxetine were augmented by chronic corticosterone administration, a model of the anxiety and depressive-like state [24,25]. In addition, alpha-calcium/calmodulin-dependent protein kinase II (α-CaMKII) heterozygous KO mice, Shunurri-2 KO mice, and pilocarpine-treated mice also exhibited enhanced adult neurogenesis concurrently with immaturity of the GCs [26-28], showing the correlation of the two adjacent cellular changes in other cases. Given the 5-HT_4_ receptor expression in mature GCs (Figure 3) and its requirement for not only GC dematuration but also increase in neurogenesis by fluoxetine (Figures 2 and 4), it is possible that dematuration-related phenotypic changes via the 5-HT_4_ receptor in mature GCs are involved in the increase in adult neurogenesis in the DG. Another possibility, however, is that chronic fluoxetine treatment affects unidentified factors via the 5-HT_4_ receptor; thus, additional mechanisms could independently regulate neurogenesis and dematuration-related phenotypic changes in the DG.

We also showed that the reduction of calbindin expression by chronic fluoxetine treatment was observed in the absence of newly-generated neurons in the DG (Figure 5). This result indicates that adult neurogenesis is not necessary for the induction of GC dematuration. However, it is noteworthy that the GC dematuration by fluoxetine was slightly attenuated in the irradiated mice compared with sham-treated mice. It has been reported that adult-born GCs modify the excitability of pre-existing mature GCs [29]. Although other effects of X-ray irradiation, such as inflammation, must be considered, it is possible that newly-generated neurons may contribute to the progression or maintenance of dematuration of GCs in a positive-feedback manner.

How can the phenotypic changes including dematuration of GCs enhance adult neurogenesis in the DG? One possibility is that there is an increase in the expression of neurogenic factors in the DG during dematuration. We showed that *Bdnf* and *Npy* expression in the DG is increased by chronic fluoxetine treatment, and that this increased expression is correlated with the progression of GC dematuration (Figure 4). The dematured state of GCs may persistently activate signaling pathways to increase the expression of these neurogenic factors. In support of this hypothesis, central NPY and BDNF administration was found to promote hippocampal neurogenesis [16,18]. It has been reported that deficiency or blockade of the BDNF receptor tropomyosin receptor kinase B (TrkB) prevents fluoxetine-mediated stimulation of mitosis in progenitor cells [17,30], suggesting the involvement of this signal in the neurogenic effect of fluoxetine. Thus, the increased expression of these neurogenic factors in the dematured DG is likely to mediate the enhancement of adult neurogenesis.

One other possibility is that an alteration occurs in the microcircuits of the hippocampus. Dematured GCs exhibit a lower threshold for action potentials, in addition to enhancement of monoamine-induced synaptic potentiation at the mossy fiber synapse [10,11]. Given that parvalbumin-positive GABAergic interneurons in the DG receive excitatory input from GCs [31], dematured GCs could affect the functions of these GABAergic interneurons in the DG. It has been reported that GABAergic signals from parvalbumin interneurons directly stimulate neural progenitors and modulate adult neurogenesis in the SGZ [32,33]. Therefore, dematured GCs may modulate adult neurogenesis via GABAergic signals in the DG.

### Behavioral effects of SSRI-induced dematuration of GCs in the DG

In this study, we suggest that dematuration and other phenotypic changes in GCs by chronic SSRI treatments may act upstream of increased neurogenesis. Therefore, it is expected that the associated phenotypic changes of GCs indirectly regulate the neurogenesis-dependent behavioral changes caused by chronic SSRI treatment. Mature GCs constitute the majority of the neuronal population in the DG and regulate hippocampal functions. Therefore, in addition to having an influence on neurogenesis-dependent functions, the dematuration of mature GCs is assumed to have a significant impact on both hippocampus-related and neurogenesis-independent behaviors. Recent reports showed that antidepressant-like behaviors resulting from chronic fluoxetine treatment were mediated by both X-ray-sensitive and -insensitive processes [9,24]. We showed that X-ray irradiation blocked adult neurogenesis, but spared the process of dematuration in GCs after chronic fluoxetine treatment (Figure 5); it is therefore likely that the behaviors blocked by X-ray irradiation are closely associated with neurogenesis. Our results additionally suggest that dematuration of GCs may contribute to X-ray insensitive antidepressant-like behaviors. However, we also observed that X-ray irradiation tends to attenuate the process of dematuration in GCs by fluoxetine treatment. Thus, caution should be exercised in interpreting the occurrence of X-ray-sensitive behaviors. We have previously reported that chronic fluoxetine treatment induced behavioral destabilization of home cage behaviors, which instability was attenuated in 5-HT4R KO mice [34]. Because a deficiency in 5-HT_4_ receptors abolished both the neurogenic and dematuration effects of fluoxetine in the DG, it is difficult to interpret which cellular changes contribute to the behavioral changes that occur in 5-HT4R KO mice. Comparison of the behavioral effects of fluoxetine between WT mice and 5-HT4R KO mice under during the blockade of adult neurogenesis by X-ray will be performed in a future study to clarify the implications of GC dematuration-associated behaviors.

## Conclusions

We demonstrated that the 5-HT_4_ receptor plays an important role in fluoxetine-induced adult neurogenesis in the DG in addition to the dematuration of GCs, and that these phenomena are closely associated. The present study suggests that chronic fluoxetine treatment induces dematuration of GCs, which in turn increases neurogenesis. An alternate possibility is that chronic fluoxetine treatment affects unidentified factors via the 5-HT_4_ receptor, which independently regulate neurogenesis and dematuration-related phenotypic changes in the DG. Our study provides new insights into the cellular mechanisms of adult neurogenesis induced by antidepressant treatment.

## Methods

### Animals

All mice were housed under standard illumination parameters with a 12-h light/dark cycle and *ad libitum* access to water and food. Male 5-HT_4_ receptor heterozygous mutant mice that had been backcrossed to the C57BL/6 J background for 10 generations were purchased from the Jackson Laboratory (Bar Harbor, ME, USA). Male (6–12 weeks old) wild-type (WT) and homozygous mutant (5HT4R KO) mice prepared by heterozygous mating were used as previously described [10,34]. For the cranial irradiation experiments, C57BL/6 N mice (5 weeks old) were purchased from the Japan SLC (Hamamatsu, Japan) and habituated for over one week before experimental procedures. All experimental procedures were approved by the Committee of Animal Research of Kyoto University Faculty of Pharmaceutical Sciences.

### Cranial irradiation

Mice (6 weeks of age) were anesthetized by pentobarbital (50 mg/kg; Kyoritsu Pharma), and exposed to cranial irradiation using a Rigaku Radiofrex 350 X-ray generator operated at 250 kV and 15 mA with a 1-mm-thick aluminum filter. X-rays at a dose of 10 Gy were delivered at a dose rate of 0.74 Gy/min. A lead shield was placed over the body of the mice except the head. Non-irradiated controls received anesthesia only. The fluoxetine treatment was started 14 days after irradiation.

### SSRI treatment

Fluoxetine hydrochloride (LKT laboratories, Inc., St. Paul, MN, USA) was intraperitoneally injected for 21 days at a dose of 22 mg/kg. The fluoxetine solution was prepared every day. Control mice were given saline. For the comparison analysis of gene expression in the DG (Figure 4D), 11 out of 21 mice were orally given fluoxetine hydrochloride dissolved in 0.2% of saccharin solution, which was applied for 28 days at a dose of 22 mg/kg. The concentration of fluoxetine was determined for individual mice based on the amount of liquid consumption quantified during the preceding 24 h period and the body weight [10]. Control mice were given water (orally).

### Measurement of tissue monoamine contents and *in vivo* microdialysis

Measurement of tissue 5-HT and 5-HIAA contents were performed as previously described with some modifications [35]. Briefly, 1-mm-thick coronal slices were prepared using a brain matrix on ice. Then, tissue blocks containing the dorsal raphe nucleus and median raphe nucleus or hippocampus were dissected, homogenized, and sonicated in 300 μL of ice-cold 0.1 M HClO_4_ containing 10 mM Na_2_S_2_O_5_ and 1 mM EDTA. Protein concentrations were measured by using the Bradford protein assay (Bio-Rad, Hercules, CA, USA). Homogenates were centrifuged at 16,000 × *g* for 15 min at 4°C, and supernatants were stored at −80°C until use. Supernatants were thawed on ice and analyzed by high-performance liquid chromatography with an electrochemical detector (HPLC-ECD) (Eicom, Kyoto, Japan). The measured 5-HT concentration was normalized against the total protein concentration. The detection limits for both 5-HT and 5-HIAA were estimated to be approximately 0.5–0.6 fmol per 25 μL of sample.

For *in vivo* microdialysis, mice were stereotactically implanted with a guide cannula (Eicom) in the ventral hippocampus according to the atlas of Franklin and Paxinos [36] with the following coordinates: anterior-posterior, −2.8 mm from the bregma; lateral, 3.0 mm from the mid line; dorsal-ventral, −2.5 mm from the skull surface. Experiments were performed 1 day after surgery in awake and freely moving mice. On the day of the experiment, a dialysis probe with a length of 1 mm (Eicom) was inserted into the guide cannula and perfused with Ringer’s solution (147 mM NaCl, 4 mM KCl, 3 mM CaCl_2_) at a flow rate of 1 μL/min for 2–3 h. After the initial perfusion period, dialysate samples were collected every 20 min. On-line quantification of 5-HT in the dialysate was accomplished by HPLC-ECD, and seven basal samples were collected before administration. Fluoxetine (22 mg/kg) or saline was then administered IP and samples were collected for an additional 120 min. The average values of the seven basal samples for each animal were defined as 100% and used for normalization. Probe placement in each mouse was histologically verified by examining the coronal brain sections after completion of the experiment.

### Immunohistochemistry

On the day following the last treatment of fluoxetine, mice intraperitoneally received BrdU (150 mg/kg, Sigma B5002). Two hours after BrdU injection, the mice were anesthetized by chloral hydrate (Nacalai Tesque, Kyoto, Japan) and transcardially perfused with cold saline followed by 4% paraformaldehyde (PFA) in 0.1 M phosphate buffer, pH 7.4. Brains were postfixed with 4% PFA overnight at 4°C, cryoprotected in 20% sucrose overnight, and stored at −80°C. Serial sections were then cut through the entire hippocampus [36] at a thickness of 30 μm with a cryostat (Leica CM3050) and stored in a non-freezing solution at −20°C until stained.

For BrdU immunostaining, every sixth section was mounted on glass slides and incubated in 0.01 M citric acid at 90°C for 5 min, denatured with 2 M hydrogen chloride at 37°C for 30 min, neutralized with 0.1 M boric acid (pH 8.5) at room temperature for 10 min, blocked in 10% equine serum in PBS containing 0.3% Triton X-100 at room temperature for 60 min, and incubated with monoclonal rat anti-BrdU (1:200; Serotec OBT0030) at room temperature overnight. For DCX and calbindin immunostaining, sections were blocked in 10% equine serum and incubated with polyclonal goat anti-DCX (1:500; Santa Cruz SC8066) or monoclonal mouse anti-calbindin D-28 K (1:3000; Sigma Aldrich, C9848). Sections were then incubated for 60 min with biotinylated goat anti-rat IgG (1:1000; Vector BA9400) as the anti-BrdU secondary antibody or biotinylated horse anti-goat IgG (1:1000; Vector BA9500) as the anti-DCX secondary antibody, or biotinylated horse anti-mouse IgG (1:1000; Vector BA2000) as the calbindin D-28 K secondary antibody, followed by incubation with ABC Vectastain Kit (Vector). Antigen detection was performed with 0.06% 3,3′-diaminobenzidine (DAB) staining and counterstaining with Nuclear Fast Red (Vector).

For immunofluorescence staining, sections were blocked in 10% equine serum and incubated overnight at 4°C with polyclonal rabbit anti-β-galactosidase (1:2000, MP, 55976) and either polyclonal goat anti-DCX (1:500; Santa Cruz SC8066), monoclonal mouse anti-calretinin (1:3000; Millipore, MAB 1568), monoclonal mouse anti-NeuN (1:500; Millipore, MAB377), or monoclonal mouse anti-calbindin D-28 K (1:3000; Sigma Aldrich, C9848). Sections were then incubated for 60 min with a secondary antibody conjugated with AlexaFluor 488 or AlexaFluor 555 (Molecular Probes).

### Quantification of BrdU-labeled cells, DCX-positive cells, and calbindin-IR

For BrdU-labeled cell quantification, a modified unbiased stereological procedure was used as previously described [37]. Sections were coded to ensure that the analysis was performed by a blind observer using a light microscope (Nikon Ecripse E200, Tokyo, Japan), and BrdU-labeled cells in the SGZ of the hippocampus were counted. Cells were included in SGZ counts if they were touching the border between the GCL and the hilus, or in the deepest layer of the GCL. If a cell was more than two cell diameters from the GCL, it was excluded. Every sixth 30-μm-thick section was evaluated throughout the hippocampus, and the sum of the cell counts was multiplied by six to provide an estimate of the total number of BrdU-labeled cells in the entire region. For DCX-positive cell quantification, 3–4 sections of the DG were photographed using a Biozero BZ-8000 microscope (Keyence Corporation, Osaka, Japan) that was fitted with a 20X objective. The boundaries of the DG were set as regions of interest (ROI), and each ROI was measured; then, DCX-positive cells were manually counted within the ROI and their number was expressed as the number of cells per square millimeter. For quantification of calbindin-IR, 2 sections of the DG were photographed in the manner described above. The pictures were converted into 16-bit gray scale pictures and the average signal intensity of calbindin-IR in the GCL region or ML region was quantified by computer-assisted image analysis (ImageJ, NIH, Bethesda, Maryland).

### *In situ* hybridization

*In situ* hybridization was performed using a digoxigenin (DIG)-labeled riboprobe as previously described [38]. A *Ht4r* cDNA template probe was cloned by PCR with gene-specific primers (Table 1), verified by sequencing, and used to produce digoxigenin (DIG)-labeled riboprobes with the DIG RNA Labeling Kit (Roche, Mannheim, Germany). Coronal brain sections (thickness, 10 μm) were cut on a cryostat, mounted onto slides, fixed in 4% PFA, acetylated, and dehydrated prior to hybridization. Sections were hybridized with the DIG-labeled riboprobe. DIG was visualized using an alkaline phosphatase-conjugated anti-DIG antibody and BM purple substrate (Roche).

**Table 1:** **List of primers used for quantitative real-time PCR analysis and for the** ***5ht4r*** **cDNA probe**

**Gene**	**Forward primer (5′ to 3′)**	**Reverse primer (5′ to 3′)**
*Bdnf*	gacaaggcaacttggcctac	actgtcacacacgctcagctc
*Npy*	ccacgatgctaggtaaca	ggaaaagtcgggagaac
*5ht4r*	gatgctaatgtgagttccaacga	gcagcagatggcgtaatacct
*18S rRNA*	gaggccctgtaattggaatgag	gcagcaactttaatatacgctattgg
*5ht4r (for in situ probe)*	ggtcaacaagccctatgctatc	taagtatcactgggctgag

### Quantitative RT-PCR

Total RNA was extracted from the DG using the ReliaPrep RNA Miniprep System (Promega), and subjected to reverse transcription with Superscript VILO (Invitrogen), and followed by real-time PCR on a LightCycler (Roche) using the Fast Start DNA Master SYBR Green I (Roche). Expression levels of target genes were normalized to the levels of 18S rRNA. Primer sequences are shown in Table 1. The specificity of each primer set was confirmed by checking the product size by gel electrophoresis.

### Statistical analyses

All data are presented as the mean ± S.E.M., and experiments with 2 groups were compared using unpaired Student’s *t*-test, whereas experiments with 4 groups were subjected to two-way ANOVA, followed by the Bonferroni *post hoc* test. A two-way repeated measure ANOVA was used for the microdialysis assay. Significance marks in figures are based on results from the *t*-test, two-way ANOVA test, and Bonferroni *post hoc* tests. The threshold for statistical significance was P < 0.05. All analyses were performed using PRISM 5 software (GraphPad, San Diego, CA).

## Abbreviations

SSRI: Selective serotonin reuptake inhibitor
GC: Granule cell
GCL: Granular cell layer
DG: Dentate gyrus
5-HT: Serotonin
5-HT4R KO: 5-HT_4_ receptor knockout
WT: Wild-type
BrdU: 5-bromodeoxyuridine
SGZ: Subgranular zone
DCX: Doublecortin
IR: Immunoreactivity
